# Signal parameter estimation of complex exponentials using fourth order statistics: additive Gaussian noise environment

**DOI:** 10.1186/s40064-015-1131-3

**Published:** 2015-07-17

**Authors:** Pradip Sircar, Mukesh K Dutta, Sudipta Mukhopadhyay

**Affiliations:** Department of Electrical Engineering, Indian Institute of Technology Kanpur, Kanpur, UP 208016 India; Department of Electronics and Electrical Communication Engineering, Indian Institute of Technology Kharagpur, Kharagpur, WB 721302 India

**Keywords:** Signal parameter estimation, Complex exponentials, Fourth order moment and cumulant, Higher order statistics

## Abstract

A novel approach based on fourth order statistics is presented for estimating the parameters of the complex exponential signal model in additive colored Gaussian noise whose autocorrelation function is not known. Monte Carlo simulations demonstrate that the proposed method performs better than an existing method which also utilizes fourth order statistics under the similar noise condition. To deal with the non-stationarity of the modeled signal, various concepts are introduced while extending the estimation technique based on linear prediction to the higher order statistics domain. It is illustrated that the accuracy of parameter estimation in this case improves due to better handling of signal non-stationarity. While forming the fourth order moment/ cumulant of a signal, the choice of the lag-parameters is crucial. It has been demonstrated that the symmetric fourth order moment/ cumulant as defined in this paper will have many desirable properties.

## Introduction

The estimation of the model parameters of a non-Gaussian stationary signal in the presence of additive Gaussian noise is conveniently carried out by a higher-order cumulant-based technique (Swami and Mendel 1991). Note that the non-Gaussian signals are frequently encountered in active sonar (Wegman et al. 1989). It is known that the cumulants of order greater than two of Gaussian processes are zero, whereas the cumulants of non-Gaussian processes carry higher order statistical information. As a consequence, the cumulant-based techniques are particularly suitable when the noise is colored Gaussian with unknown power spectral density (PSD).

A transient signal is traditionally represented by the parametric model consisting of a sum of complex exponentials, and the model parameters are estimated by Prony’s method (Henderson 1981; Kumaresan and Tufts 1982). Parameter estimation of the sum of complex exponential signals is closely related to the problem of system identification, because the impulse response of a linear time-invariant (LTI) system with distinct poles has the similar parametric form. Moreover, the sum of complex exponential signals provides a more general model than the sum of complex sinusoidal signals. In this way, the parametric representation can be extended from a stationary signal to a non-stationary signal, as explained in the sequel. When the signal derived from a multicomponent complex exponential random process is corrupted with white noise, an extension of Prony’s method to the second-order statistics domain can provide better accuracy of estimation of model parameters (Cadzow and Wu 1987; Sircar and Mukhopadhyay 1995). It should be mentioned, however, that special care is to be taken in the extension of the estimation technique to the higher order statistics domain because the signal being modeled is non-stationary in nature (Sircar and Mukhopadhyay 1995), and the cumulants of a non-stationary process are, in general, time-dependent.

In the presence of additive noise which is colored Gaussian with unknown autocorrelation sequence, an estimation technique utilizing the fourth order moment/ cumulant sequence of the modeled signal will be desirable. In this paper, several new concepts related to higher order statistics, which can be implemented for estimating the parameters of a transient signal in noise are presented. It is demonstrated how the concept of accumulated moment can be introduced in the context of the non-stationarity of the modeled signal (Sircar and Mukhopadhyay 1995). The case where a single data record is available is studied separately because a replacement of the ensemble average by a temporal average will alter the result for this type of signal.

While forming the higher order moments and cumulants, the selection of the definition to be used and the choice of the lag-parameters in the definition are crucial (Swami and Mendel 1991). It is demonstrated that the moments and cumulants defined and used in the proposed methods will have many desirable properties. Moreover, it is shown by Monte Carlo simulation that the method presented here will perform better than another method (Papadopoulos and Nikias 1990), even though both of the methods utilize the fourth order statistics (FOS) of the sampled signal.

In the present work, we consider fourth order moment/ cumulant of a non-stationary signal for parameter estimation. The FOS has been used for parameter estimation with multiplicative noise model (Swami 1994), for detection of transient acoustic signals (Ravier and Amblard 1998), and for blind identification of non-minimum phase finite impulse response (FIR) system (Belouchrani and Derras 2000). In recent time, the FOS has been applied for blind source identification where number of sources exceeds number of sensors (Ferréol et al. 2005), for underdetermined independent component analysis (ICA) (Zarzoso et al. 2006; Lathauwer et al. 2007), and for ICA with multiplicative noise (Blanco et al. 2007). Lately, it has been shown that the FOS-based direction-of-arrival (DOA) estimation of non-Gaussian source has many advantages compared to the subspace based methods (Zeng et al. 2009).

In the continuation work, the method based on fourth-order moment/ cumulant, as developed in this paper, has been applied for parameter estimation of non-stationary signals in multiplicative and additive noise (Gaikwad et al. 2015). We assume that the additive noise is Gaussian and the signal component comprising of the multiplicative noise is non-Gaussian. It has been demonstrated in the accompanying paper that the concept of accumulated moment, as presented here, can be extended to the concept of accumulated cumulant while estimating parameters of some non-stationary signals in multiplicative noise (Gaikwad et al. 2015).

The paper is organized in the following way: The accumulated fourth order moment for a non-stationary signal is defined in "Accumulated fourth order moments". The case of signal corrupted with noise is considered in "Noise-corrupted signal case", where the fourth order cumulant of the signal is derived. In "Deterministic signal case", the case of deterministic signal is considered, and the fourth order time cumulant of the signal is defined. The simulation study is presented in "Simulation study", and the conclusion is drawn in "Conclusion".

## Accumulated fourth order moments

The discrete-time random signal model *X*[*n*] consisting of *M* complex exponential signals with the circular frequencies ${\omega }_i$, damping factors ${\alpha }_i$ (negative), amplitudes $A_i$ and phase ${\phi }_i$ is expressed as

1 $$\begin{aligned} X[n] = \sum _{i=1}^M A_i \exp (j\phi _i) \exp \left[ (\alpha _i + j\omega _i)n\right] \end{aligned}$$

where $A_{ci} = A_i \exp (j{\phi }_i)$ are the complex amplitudes, and $z = \exp ({\alpha }_i + j{\omega }_i)$ are the modes of the signal. For real signals, $z_i$ and $A_{ci}$ must occur in complex conjugate pairs. It is assumed that the phase ${\phi }_i$ are independent and identically distributed (i.i.d.) with the uniform probability density function (PDF) over $[0,2\pi )$.

By utilizing the linear prediction (LP) model, the optimal forward predictor is

2 $$\begin{aligned} \hat{X}[n] = - \sum _{\ell =1}^M a_{\ell } X[n-\ell ] \end{aligned}$$

and the optimal backward predictor is given by

3 $$\begin{aligned} \hat{X}[n] = - \sum _{\ell =1}^M a_{\ell }^{\star } X[n+\ell ] \end{aligned}$$

where $(\star )$ denotes complex conjugation (Kumaresan and Tufts 1982). The coefficients $a_{\ell }$ of the predictor error filter (PEF) $\mathcal{{A}}(z)$ are related to the modes $z_i$ as follows

4 $$\begin{aligned} \mathcal{{A}}(z) = 1 + \sum _{\ell =1}^M a_{\ell } z^{- \ell } = \prod _{i=1}^M (1 - z_i z^{-1}) \end{aligned}$$

The forward and backward prediction errors, $E_f[n]$ and $E_b[n]$ respectively, are expressed as

5 $$\begin{aligned} E_f[n] = \sum _{\ell =0}^M a_{\ell } X[n-\ell ] \end{aligned}$$

6 $$\begin{aligned} E_b[n] = \sum _{\ell =0}^M a_{\ell }^{\star } X[n+\ell ] \end{aligned}$$

with $a_0 = 1$. By minimizing the forward prediction error power estimated over a fixed frame between properly selected $n_1$ and $n_2$,

7 $$\begin{aligned} P_f = \sum _{n=n_1}^{n_2} \mathcal{{E}} \left\{ E_f^2 [n] \right\} \end{aligned}$$

where $\mathcal{{E}}$ denotes the expectation operator, one obtains the covariance method as follows (Kumaresan and Tufts 1982; Sircar and Mukhopadhyay 1995),

8 $$\begin{aligned} \frac{\partial P_f}{\partial a_m} = 0 \qquad {\Longrightarrow }\nonumber \\ \sum _{\ell =0}^M a_{\ell } \sum _{n=n_1}^{n_2} \mathcal{{E}} \left\{ X^{\star }[n-m] X[n-\ell ] \right\} = 0 \quad {\mathrm{for}}\,\, m = 0,1,\ldots ,M \end{aligned}$$

As a better alternative, the forward prediction error estimated over a moving frame with fixed length,

9 $$\begin{aligned} P_{fm} = \sum _{n=n_1+m}^{n_2+m} \mathcal{{E}} \left\{ E_f^2 [n] \right\} \end{aligned}$$

can be minimized, and this procedure leads to the improved covariance method (Sircar and Mukhopadhyay 1995),

10 $$\begin{aligned} \sum _{\ell =0}^M a_{\ell } \sum _{n=n_1+m}^{n_2+m} \mathcal{{E}} \left\{ X^{\star }[n-m] X[n-\ell ] \right\} = 0 \qquad \text{ or, } \nonumber \\ \sum _{\ell =0}^M a_{\ell } \sum _{n=n_1}^{n_2} \mathcal{{E}} \left\{ X^{\star }[n] X[n+(m-\ell )] \right\} = 0 \quad \text{ for }\,\, m = 0,1,\ldots ,M \end{aligned}$$

which is computationally efficient due to the Toeplitz structure of the system matrix.

In a similar fashion, the minimization of the estimate of backward prediction error power,

11 $$\begin{aligned} P_b = \sum _{n=n_1}^{n_2} \mathcal{{E}} \left\{ E_b^2 [n] \right\} \end{aligned}$$

leads to the backward covariance method given by

12 $$\begin{aligned} \sum _{\ell =0}^M a_{\ell }^{\star } \sum _{n=n_1}^{n_2} \mathcal{{E}} \left\{ X^{\star }[n+m] X[n+\ell ] \right\} = 0 \quad \text{ for }\,\, m = 0,1,\ldots ,M \end{aligned}$$

and for computational efficiency, the minimization of the estimate of error power

13 $$\begin{aligned} P_{bm} = \sum _{n=-n_1-m}^{-n_2-m} \mathcal{{E}} \left\{ E_b^2 [n] \right\} \end{aligned}$$

results in the improved backward covariance method as shown below (Sircar and Mukhopadhyay 1995),

14 $$\begin{aligned} \sum _{\ell =0}^M a_{\ell }^{\star } \sum _{n=n_1}^{n_2} \mathcal{{E}} \left\{ X^{\star }[-n] X[-n+(\ell -m)] \right\} = 0 \quad {\mathrm{for}}\,\,m = 0,1,\ldots ,M \end{aligned}$$

or interchanging $\ell$ and *m* it becomes,

15 $$\begin{aligned} \sum _{m=0}^M a_{m}^{\star } \sum _{n=n_1}^{n_2} \mathcal{{E}} \left\{ X^{\star }[-n] X[-n+(m-\ell )] \right\} = 0 \quad \text{ for }\,\,\ell = 0,1,\ldots ,M \end{aligned}$$

where the system matrix once again gets the Toeplitz structure.

To understand the effect of summation over *n* between properly selected $n_1$ and $n_2$ used in (10) and (15), we compute the time-varying autocorrelation functions (ACFs) of *X*[*n*] considered to be a random sequence due to its random phase ${\phi }_i$ (1),

16 $$\begin{aligned} R_{xx}[n,k] = \mathcal{{E}} \left\{ X^{\star }[n] X[n+k] \right\} = \sum _{i=1}^M A_i^2 exp(2 \alpha _i n) z_i^k \end{aligned}$$

17 $$\begin{aligned} R_{xx}[-n,k] = \mathcal{{E}} \left\{ X^{\star }[-n] X[-n+k] \right\} = \sum _{i=1}^M A_i^2 exp(-2 \alpha _i n) z_i^k \end{aligned}$$

where it is assumed that the phase $\left\{ {\phi }_i; i=1,\ldots ,M\right\}$ are i.i.d. with PDF $U[0,2\pi )$.

The accumulated ACFs $Q_{xx}^+ [k]$ and $Q_{xx}^- [k]$ can then be computed by taking summation over *n* as shown below,

18 $$\begin{aligned} Q_{xx}^+ [k] = \sum _{n=n_1}^{n=n_2} R_{xx}[n,k] = \sum _{i=1}^M B_i z_i^k\end{aligned}$$

19 $$\begin{aligned} Q_{xx}^- [k] = \sum _{n=n_1}^{n=n_2} R_{xx}[-n,k] = \sum _{i=1}^M D_i z_i^k \end{aligned}$$

where $$B_i = A_i^2 \sum _{n=n_1}^{n_2} exp(2 \alpha _i n)$$ and, $$D_i = A_i^2 \sum _{n=n_1}^{n_2} exp(-2 \alpha _i n)$$.

It should be noted that the accumulated ACFs are functions of lag *k* in a similar way as the discrete-time random process *X*[*n*] is function of time *n*. Consequently, the accumulated ACFs will satisfy the linear prediction equations in lag $k (= m-\ell )$ (10, 15). Note here that for a wide-sense stationary (WSS) process the ACF is time-invariant, and it satisfies the prediction equation in lag *k*. Apparently, the role of accumulated second-order moment for the non-stationary process is quite similar to the role of second-order moment for a WSS process (Sircar and Mukhopadhyay 1995).

We now extend the concept of accumulated moment to higher-order statistics domain. We define the symmetric fourth order moment $R_{4X} [n,k]$ of the sequence *X*[*n*] as follows,

20 $$\begin{aligned} R_{4X} [n,k] = \mathcal{{E}} \left\{ X^{\star }[n] X[n+k] X^{\star }[-n] X[-n+k] \right\} \end{aligned}$$

Substituting for $X^{\star }[n]$, $X[n+k]$, $X^{\star }[-n]$ and $X[-n+k]$ from (1), and evaluating the expectation we get,

21 $$\begin{aligned} R_{4X}[n,k]&= \mathcal{{E}}\Bigg \{\sum _{i=1}^M A_i e^{s_i^{\star }n} e^{-j\phi _i} \sum _{u=1}^M A_u e^{s_u(n+k)} e^{j\phi _u} \nonumber \\&\quad \quad \sum _{l=1}^M A_l e^{-s_l^{\star }n} e^{-j\phi _l} \sum _{v=1}^M A_v e^{s_v(-n+k)} e^{j\phi _v} \Bigg \} \nonumber \\&= \sum _{u=1}^M \sum _{v=1}^M A_u^2 A_v^2 e^{(s_u^{\star }+s_u-s_v^{\star }-s_v)n} e^{(s_u+s_v)k} \nonumber \\&\quad + \sum _{u=1}^M \sum _{v=1}^M A_u^2 A_v^2 e^{(s_v^{\star }+s_u-s_u^{\star }-s_v)n} e^{(s_u+s_v)k} \nonumber \\&\quad - \sum _{u=1}^M A_u^4 e^{(s_u^{\star }+s_u-s_u^{\star }-s_u)n} e^{2 s_u k} \end{aligned}$$

where the complex parameter $s_i = {\alpha }_i + j{\omega }_i$ is related to the mode $z_i$ as $z_i = e^{s_i}$ , and the following result of expectation is used :

22 $$\begin{aligned} \mathcal{{E}} \left\{ e^{j(\phi _i + \phi _u - \phi _l + \phi _v)} \right\} &= {} 1 \quad \text{ when } i=u, l=v, \hbox {and}\, u{\ne }v \nonumber \\ & = {} 1 \quad \text{ when } \, i=v, l=u, \hbox {and}\, u{\ne }v \nonumber \\ & = {} 1 \quad \text{ when }\, i=u=l=v \nonumber \\ & = {} 0 \quad \text{ otherwise } \end{aligned}$$

Note that in (21), the third case ($i = u = l = v$) is included twice in the first two summations, and that is why the last summation is subtracted and not added as desired. It is to be pointed out that the symmetric fourth order moment as it is defined here becomes independent of time for the case of a single-component signal even when the signal is non-stationary in nature.

Further simplification of (21) yields

23 $$\begin{aligned} R_{4X}[n,k]&= \sum _{u=1}^M \sum _{v=1}^M A_u^2 A_v^2 e^{2(\alpha _u - \alpha _v)n} e^{(s_u+s_v)k} \nonumber \\&\quad + \sum _{u=1}^M \sum _{v=1}^M A_u^2 A_v^2 e^{2j(\omega _u - \omega _v)n} e^{(s_u+s_v)k} - \sum _{u=1}^M A_u^4 e^{2s_uk} \end{aligned}$$

Note that the fourth order moment $R_{4X}[n,k]$ is a function of both time and lag variables. At this point we introduce the concept of accumulated moment (Sircar and Mukhopadhyay 1995), and compute the accumulated fourth order moment $Q_{4X}$ by taking summation over *n* between properly selected $n_1$ and $n_2$ as shown below,

24 $$\begin{aligned} Q_{4X}[k] = \sum _{n=n_1}^{n_2} R_{4X}[n,k] = \sum _{u=1}^M \sum _{v=1}^M E_{uv} e^{(s_u+s_v)k} \nonumber \\ + \sum _{u=1}^M \sum _{v=1}^M F_{uv} e^{(s_u+s_v)k} - \sum _{u=1}^M G_u e^{2s_uk} \end{aligned}$$

where $E_{uv}=A_u^2 A_v^2 \sum _{n=n_1}^{n_2} e^{2(\alpha _u-\alpha _v)n}$, $F_{uv}=A_u^2 A_v^2 \sum _{n=n_1}^{n_2} e^{2j(\omega _u-\omega _v)n}$, and $G_u=A_u^4 \left( n_2 - n_1 + 1 \right)$. Written in compact form (24) becomes

25 $$\begin{aligned} Q_{4X}[k]= & {} \sum _{u=1}^M \sum _{v=1}^M H_{uv} e^{(s_u+s_v)k} \nonumber \\= & {} \sum _{u=1}^M \sum _{v=1}^M H_{uv} \left( z_u z_v \right) ^k \end{aligned}$$

$$\begin{aligned} \text{ where } \ H_{uv} & = {} E_{uv} + F_{uv} \quad \qquad \text{ when }\, u{\ne }v \\ & = {} E_{uu} + F_{uu} - G_u \quad \text{ otherwise } \end{aligned}$$

and $$\quad z_u = e^{s_u} \text{, }\,\, z_v = e^{s_v}$$.

It is apparent from (25) that the accumulated fourth order moment (AFOM) is time-invariant as desired. Remember that the underlying process is non-stationary in nature. Moreover, it is to be noted that the AFOM sequence will satisfy the linear prediction equations in lag *k* (Sircar and Mukhopadhyay 1995). However, the modes of the AFOM sequence $Q_{4X} [k]$ are not the same modes as those of the discrete-time signal *X*[*n*]. The modes of AFOM sequence are, indeed, the square and product modes. For every mode $z = |z|\angle {\theta }$ in *X*[*n*], the square mode $z^2 = |z|^2\angle {2\theta }$ is included in $Q_{4X} [k]$. Again, for every pair of modes $z_1 = |z_1|\angle {\theta _1}$ and $z_2 = |z_2|\angle {\theta _2}$ in the signal, the product mode $z_1 z_2 = |z_1| |z_2| \angle {(\theta _1 + \theta _2)}$ is also included in the AFOM sequence. Clearly, if there are *M* modes in the original signal, the AFOM sequence will contain a total $L = M + {}^M C_2 = M(M+1)/2$ modes. Accordingly, the sequence will satisfy the linear prediction equations of order L or higher (Deprettere 1988); and once the modes of the AFOM sequence are extracted, there will be redundancy of information for computing the modes of the underlying signal.

## Noise-corrupted signal case

When the signal is corrupted with additive noise, the observed sequence *Y*[*n*] is represented as

26 $$\begin{aligned} Y[n] = X[n] + W[n] \end{aligned}$$

where *W*[*n*] is the random noise whose statistics may not be completely known. In general, the moments of *X*[*n*] and *Y*[*n*] will be different. In all cases, it is assumed that the noise sequence is zero-mean, and the signal and noise sequences are uncorrelated.

It is easy to show that the symmetric fourth order moments of the noisy and noiseless signals are same, that is, $R_{4Y} [n,k] = R_{4X} [n,k]$, with an additional condition that the noise sequence is white. This case together with the complementary case where the noise correlation is known, however, can be adequately dealt with in the second-order statistics domain (Cadzow and Wu 1987; Sircar and Mukhopadhyay 1995). Therefore, these situations are not considered here any further.

Instead, we consider the case with an additional condition that the noise sequence is colored Gaussian of unknown PSD. It is not difficult to show that the fourth order cumulant $C_{4Z} [n,k]$ of the sequence *Z*[*n*] which may be either *X*[*n*] or *Y*[*n*], does not change when the noiseless signal becomes noisy, that is, $C_{4X} [n,k] = C_{4Y} [n,k]$, where we define the symmetric fourth-order cumulant as

27 $$\begin{aligned} C_{4Z}[n,k]&=\mathcal{{E}}\left\{ Z^{\star }[n] Z[n+k] Z^{\star }[-n] Z[-n+k] \right\} \nonumber \\&\quad - \mathcal{{E}}\left\{ Z^{\star }[n] Z[n+k] \right\} \mathcal{{E}}\left\{ Z^{\star }[-n] Z[-n+k] \right\} \nonumber \\&\quad - \mathcal{{E}}\left\{ Z^{\star }[n] Z^{\star }[-n] \right\} \mathcal{{E}}\left\{ Z[n+k] Z[-n+k] \right\} \nonumber \\&\quad - \mathcal{{E}}\left\{ Z^{\star }[n] Z[-n+k] \right\} \mathcal{{E}}\left\{ Z^{\star }[-n] Z[n+k] \right\} \end{aligned}$$

for the zero-mean complex process *Z*[*n*] (Swami and Mendel 1991).

Therefore, it suffices to investigate the noiseless signal case, because the same result can be used when the signal becomes noise-corrupted. The fourth order cumulant $C_{4X} [n,k]$ for the noiseless case and for the choice of collection of arguments as proposed here is represented as

28 $$\begin{aligned} C_{4X}[n,k]&= R_{4X}[n,k] \nonumber \\&\quad - \mathcal{{E}}\left\{ \sum _{i=1}^M A_i e^{s_i^{\star }n} e^{-j\phi _i} \sum _{u=1}^M A_u e^{s_u(n+k)} e^{j\phi _u} \right\} \nonumber \\&\quad \times \mathcal{{E}}\left\{ \sum _{l=1}^M A_l e^{-s_l^{\star }n} e^{-j\phi _l} \sum _{v=1}^M A_v e^{s_v(-n+k)} e^{j\phi _v} \right\} \nonumber \\&\quad - \mathcal{{E}}\left\{ \sum _{i=1}^M A_i e^{s_i^{\star }n} e^{-j\phi _i} \sum _{l=1}^M A_l e^{-s_l^{\star }n} e^{-j\phi _l} \right\} \nonumber \\&\quad \times \mathcal{{E}}\left\{ \sum _{u=1}^M A_u e^{s_u(n+k)} e^{j\phi _u} \sum _{v=1}^M A_v e^{s_v(-n+k)} e^{j\phi _v} \right\} \nonumber \\&\quad - \mathcal{{E}}\left\{ \sum _{i=1}^M A_i e^{s_i^{\star }n} e^{-j\phi _i} \sum _{v=1}^M A_v e^{s_v(-n+k)} e^{j\phi _v} \right\} \nonumber \\&\quad \times \mathcal{{E}}\left\{ \sum _{l=1}^M A_l e^{-s_l^{\star }n} e^{-j\phi _l} \sum _{u=1}^M A_u e^{s_u(n+k)} e^{j\phi _u} \right\} \end{aligned}$$

Note that the fourth order moment $R_{4X}[n,k]$ is computed earlier, and

29 $$\begin{aligned} \text{ the } \text{ second } \text{ term }= - \sum _{u=1}^M \sum _{v=1}^M A_u^2 A_v^2 e^{(s_u^{\star }+s_u-s_v^{\star }-s_v)n} e^{(s_u+s_v)k} \end{aligned}$$

where we use the result

30 $$\begin{aligned} \mathcal{E} \left\{ e^{j(- \phi _i + \phi _u)} \right\}= & {} 1 \quad \text{ for }\,\, i=u \nonumber \\= & {} 0 \quad \text{ otherwise } \end{aligned}$$

On observation that the third term in (28) is identically zero, and that

31 $$\begin{aligned} \text{ the } \text{ fourth } \text{ term } = - \sum _{u=1}^M \sum _{v=1}^M A_u^2 A_v^2 e^{(s_u^{\star }-s_u-s_v^{\star }+s_v)n} e^{(s_u+s_v)k} \end{aligned}$$

the expression for the fourth order cumulant can be rewritten as,

32 $$\begin{aligned} C_{4X}[n,k]&= R_{4X}[n,k] - \sum _{u=1}^M \sum _{v=1}^M A_u^2 A_v^2 e^{2(\alpha _u-\alpha _v)n} e^{(s_u+s_v)k} \nonumber \\&\quad - \sum _{u=1}^M \sum _{v=1}^M A_u^2 A_v^2 e^{2j(\omega _u-\omega _v)n} e^{(s_u+s_v)k} \end{aligned}$$

after simplification. Substituting $R_{4x} [n,k]$ from (23) and canceling terms, (32) reduces to

33 $$\begin{aligned} C_{4X}[n,k] = - \sum _{u=1}^M A_u^4 e^{2s_uk} = C_{4X}[k] \end{aligned}$$

This result is remarkable, because it tells us that for the particular choice of arguments as presented in this paper, the symmetric fourth order cumulant is independent of time, that is, $C_{4X}[n,k]=C_{4X}[k]$, although the underlying transient signal is non-stationary in nature.

Note that the fourth order cumulant will satisfy the *M*-order linear prediction equations in lag exactly in the same way as the signal does that in time (Sircar and Mukhopadhyay 1995; Deprettere 1988). Moreover, the modes of the cumulant are the square modes of the signal, and the product modes as defined earlier are missing.

## Deterministic signal case

In last two sections we considered the case of random signal in some detail. In this section, a treatise is presented for the non-random signal. Although the observed sequence can be thought of as a sample of some discrete-time random process, any replacement of ensemble average by temporal average will likely produce a different set of results because the underlying signal is non-stationary in nature.

We consider a finite-length sampled set of the signal, and compute the $\tilde{C}$-sequence which is defined as follows,

34 $$\begin{aligned} \tilde{C}[k]&= \frac{1}{n_2-n_1+1} \sum _{n=n_1}^{n_2} \bar{X}^{\star }[n] \bar{X}[n+k] \bar{X}^{\star }[-n] \bar{X}[-n+k] \nonumber \\&\quad - \frac{1}{(n_2-n_1+1)^2} \sum _{n=n_1}^{n_2} \bar{X}^{\star }[n] \bar{X}[n+k] \sum _{m=n_1}^{n_2} \bar{X}^{\star }[-m] \bar{X}[-m+k] \nonumber \\&\quad - \frac{1}{(n_2-n_1+1)^2} \sum _{n=n_1}^{n_2} \bar{X}^{\star }[n] \bar{X}^{\star }[-n] \sum _{m=n_1}^{n_2} \bar{X}[m+k] \bar{X}[-m+k] \nonumber \\&\quad - \frac{1}{(n_2-n_1+1)^2} \sum _{n=n_1}^{n_2} \bar{X}^{\star }[n] \bar{X}[-n+k] \sum _{m=n_1}^{n_2} \bar{X}^{\star }[-m] \bar{X}[m+k] \nonumber \\ \end{aligned}$$

where $\bar{X}[n] = X[n] - X_0$, $X_0$ being the mean of the finite-length data record. Note that $X_0$ is usually small but may not be identically zero. It is to be pointed out that (34) is very similar to (27), except that the time-averaging over properly selected $n_1$ and $n_2$ replaces the expectation operation. The choice of $n_1$ and $n_2$ should be such that there is no running off the ends of data record (Sircar and Mukhopadhyay 1995).

On substitution of (1) and simplification, the terms of (34) reduce to the general form as shown below :

35 $$\begin{aligned}&\frac{1}{n_2-n_1+1} \sum _{n=n_1}^{n_2} \bar{X}^{\star }[n] \bar{X}[n+k] \bar{X}^{\star }[-n] \bar{X}[-n+k] \nonumber \\ &\quad = {} \sum _{u=1}^M \sum _{v=1}^M t_{11}[u,v] e^{(s_u+s_v)k} + \sum _{u=1}^M t_{12}[u] e^{s_uk} + t_{13} \nonumber \\&\quad \quad- \frac{1}{(n_2-n_1+1)^2} \sum _{n=n_1}^{n_2} \bar{X}^{\star }[n] \bar{X}[n+k] \sum _{m=n_1}^{n_2} \bar{X}^{\star }[-m] \bar{X}[-m+k] \nonumber \\ & \quad = {} - \sum _{u=1}^M \sum _{v=1}^M t_{21}[u,v] e^{(s_u+s_v)k} - \sum _{u=1}^M t_{22}[u] e^{s_uk} - t_{23} \nonumber \\&\qquad \qquad \qquad \qquad \qquad \vdots \end{aligned}$$

where each coefficient $t_{\ell 1}$ is made independent of time *n* (and *m*), indices *i* and *l* [see (28)] by taking summation over respective variables. Similarly, each of $t_{\ell 2}$ is independent of all variables except *u*, and every $t_{\ell 3}$ is made independent of all six variables by summation. It is to be pointed out that if the mean $X_0 = 0$, the coefficients $t_{\ell 2}$ and $t_{\ell 3}$ will be identically zero. In this case, each of $t_{\ell 1}$ will again be a non-zero factor.

Combining all four terms of (35), (34) is rewritten as

36 $$\begin{aligned} \tilde{C}[k] = \sum _{u=1}^M \sum _{v=1}^M T_1[u,v] e^{(s_u+s_v)k} + \sum _{u=1}^M T_2[u] e^{s_uk} + T_3 \end{aligned}$$

where $T_1 = t_{11} - t_{21} - t_{31} - t_{41}$, etc., and $T_2$, $T_3$ are non-zero only when $X_0$ is not equal to zero. Note that the forms of (25) and (36) will be similar when $X_0 = 0$. Another point worth mentioning here is that $T_2$ will have $X_0$ (or $X_0^{\star }$) as a factor, whereas $T_3$ will involve higher power terms of $X_0$ (or $X_0^{\star }$). As a consequence, when $X_0$ is small, as will be the case here, $T_3$ can be dropped from (36) retaining $T_2$ for small but significant value. Rewriting (36) for small $X_0$, one obtains

37 $$\begin{aligned} \tilde{C}[k] = \sum _{u=1}^M \sum _{v=1}^M T_1[u,v] e^{(s_u+s_v)k} + \sum _{u=1}^M T_2[u] e^{s_uk} \end{aligned}$$

Note that even if $T_3$ is not negligible, the mode corresponding to the dropped term from (37) is real unity, which can be easily identified and discarded.

Comparing (25) and (37), it can be observed that the $\tilde{C}$-sequence consists of the square and product modes of the signal, together with the original signal modes. If there are *M* modes in the sampled signal, the number of modes in the $\tilde{C}$-sequence will be $L = M + M(M+1)/2 = M(M+3)/2 \,\,$. Consequently, the sequence will satisfy the linear prediction equations of order more than *L*. Remember that the unity mode may also be present.

When the signal is corrupted with noise, the $\tilde{C}$-sequence which may be termed as the fourth order time cumulant (FOTC), will deviate. However, it is likely that this deviation will be small when the superimposed noise is zero-mean Gaussian and uncorrelated with the signal. Remember that we are computing the time-average here.

## Simulation study

The discrete-time signal $X[\tilde{n}]$ of (1) is sampled at $\tilde{n} = 0,1,\ldots ,2N+1$ with $N = 50$ utilising the following parameters, $M = 2$, $A_1 = 1.0$, $\alpha _1 = - 0.02$, $\omega _1 = 0.3$, $\phi _1 = 0$, $A_2 = 1.0$, $\alpha _2 = - 0.01$, $\omega _2 = 0.5$, $\phi _2 = \pi /4$. The time-origin is then shifted by making $n = \tilde{n}-N$ such that the sequence $\{X[n]; n = -N,\ldots ,0,\ldots ,N\}$ is now available for processing. The shifting does not change the modes of the signal, only the complex amplitudes of the components change to new values [refer to (1)].

Next, the zero-mean complex colored Gaussian noise is added to the signal setting the signal-to-noise ratio (SNR) at various designated levels. The colored noise is generated by passing a complex white Gaussian process through a coloring filter as mentioned in (Papadopoulos and Nikias 1990). The fourth order time cumulants $\tilde{C}[k]$ are then computed by (34) for the lags $k = -K,\ldots ,0,\ldots ,K$ with $K = 25$, that is a total of 51 points. The time-frame is chosen with $n_1 = 8$ and $n_2 = 25$. The corresponding frame-length $(n_2-n_1+1)$ lies between *N* / 3 and 2*N* / 5 ensuring optimal performance (Hua and Sarkar 1990).

There are $L = 5$ modes in the $\tilde{C}$-sequence. We use backward linear prediction model with the extended model order $J=17$ and the singular value decomposition based total least squares (SVD-TLS) technique for extraction of the *L* signal $(1/z_i^{\star })$ and $(J-L)$ noise modes (Kumaresan and Tufts 1982; Cadzow and Wu 1987; Deprettere 1988). Remember that the modes of the $\tilde{C}$-sequence are the square, product and original modes of the sampled sequence *X*[*n*].

For the purpose of comparison, the same noise-corrupted data record is processed employing the fourth order cumulant (FOC) method as proposed in (Papadopoulos and Nikias 1990). The extended model order used is same in each of the cases, which ensures that the computational efforts for extracting modes are roughly equal for the methods.

**Figure 1:**
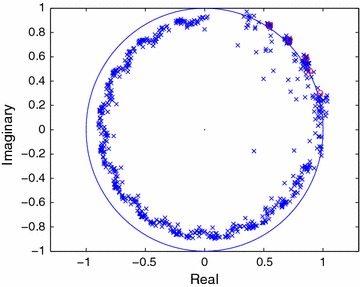
Estimated poles of the FOTC sequence in colored noise (SNR = 10 dB).

**Figure 2:**
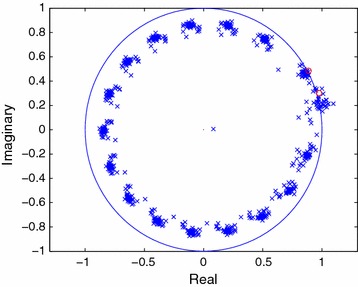
Estimated poles of the FOC sequence in colored noise (SNR = 10 dB).

Setting the SNR level at 10 dB in each trial, the modes are computed and plotted for 40 independent noise sequences employing the FOTC method as proposed in this paper and the FOC method as presented in (Papadopoulos and Nikias 1990). The plots are shown in Figures 1 and 2 where the actual modes ($1/z_i^{\star }$) of the respective sequences are shown by circles.

It is seen in Figure 1 that for the $\tilde{C}$-sequence, the square and product modes are accurately determined, whereas the original signal modes are often undetected at this SNR level. Moreover, it is found that the square modes are estimated with better accuracy than the product modes. Note that it is easy to identify and utilize the square modes of the signal for estimating the modes of the underlying signal. First the square-roots of all the estimated modes of the $\tilde{C}$-sequence are to be computed, then the complex amplitudes are to be estimated for all these computed modes utilizing the original signal sequence (Cadzow and Wu 1987). Selecting the original signal modes for *M* larger amplitudes will then be relatively straightforward. Note that in this way, the signal modes will be determined utilizing the square modes of the $\tilde{C}$-sequence. Thus, it should be the strategy that the modes of the underlying signal are to be determined utilizing the accurate estimates of the square modes of the $\tilde{C}$-sequence, because the estimates of the signal modes of the $\tilde{C}$-sequence may be inaccurate when the signal samples are corrupted with noise.

**Figure 3:**
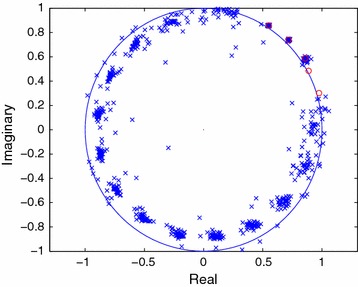
Estimated poles of the FOTC sequence in white noise (SNR = 10 dB).

**Figure 4:**
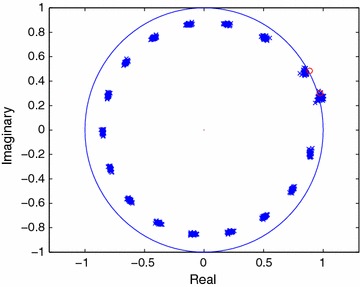
Estimated poles of the FOC sequence in white noise (SNR = 10 dB).

In Figure 2, the estimates of the signal modes are shown for 40 independent trials when the FOC method is employed. The effort here is directed towards finding the fourth-order cumulant which will have the same modes $(1/z_i^{\star })$ as those of the signal. In Figures 3 and 4, the estimated modes of the $\tilde{C}$-sequence and those of the signal are plotted for 40 independent trials when the noise sequence is complex Gaussian, zero mean and white. The comparisons between Figures 1, 3 and Figures 2, 4 reveal how the performance of a particular method changes when the noise sequence becomes white or colored.

**Figure 5:**
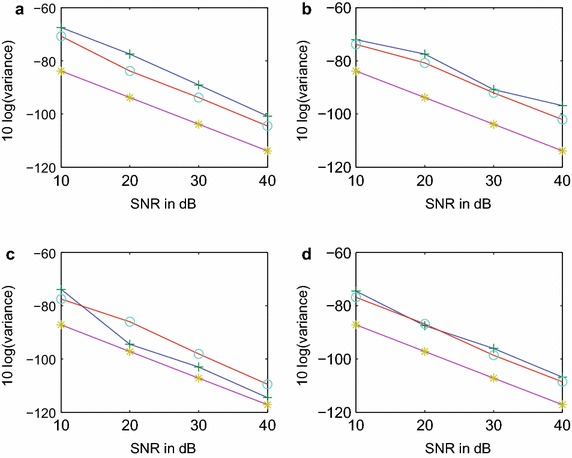
Error-variance of estimation for* α* and $\omega$ parameters in colored noise (circle-FOTC method, plus-FOC method, star-CR bound). **a** α_1_-variance, **b** *ω*_1_-variance, **c** α_2_-variance, **d** *ω*_2_-variance.

**Figure 6:**
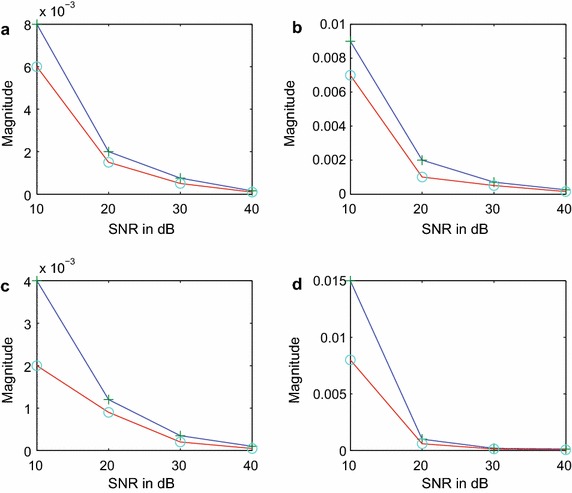
Bias-magnitude of estimation for *α* and $\omega$ parameters in colored noise (circle-FOTC method, plus-FOC method). **a** α_1_-bias, **b** *ω*_1_-bias, **c** α_2_-bias, **d** *ω*_2_-bias.

The comparison of performances of the two methods is then demonstrated by the Monte Carlo simulation. The zero-mean colored Gaussian noise is mixed with the signal samples setting the SNR levels at 10, 20, 30 and 40 dB. The estimates of the $s_i$-parameters are obtained for 500 independent runs employing the two methods for various SNRs. The error-variances and bias-magnitudes are computed for the estimates of $\alpha _i$ and $\omega _i$-values for each SNR level. The computed values are plotted in Figures 5a–d and 6a–d. The SNR versus error-variance plots also include the Cramer-Rao (CR) bounds for the estimated parameters for comparison (Hua and Sarkar 1990), see Figure 5a–d.

It can be observed from Figure 5a–d that the performance of the proposed FOTC method in regard to the variance of estimate is either comparable or somewhat better than the performance of the FOC method developed in (Papadopoulos and Nikias 1990). However when we extend the comparison to the bias of estimate, it is seen from Figure 6a–d that the performance of the proposed method is much better than the performance of the FOC method. The comparisons of Figures 5 and 6 clearly show the advantage of the proposed FOTC method over the existing FOC method for better accuracy of estimation.

## Conclusion

In this paper, a new method based on utilizing the computed fourth order time cumulants of a transient signal is presented for estimating the parameters of the modeled signal. The proposed method performs better than the FOC method developed in (Papadopoulos and Nikias 1990), even though the latter is another method based on the fourth order statistics.

It is known that the fourth order moments and cumulants can be defined and evaluated in a variety of ways (Swami and Mendel 1991). It is note-worthy that when the fourth order moments/ cumulants are utilized in the problem of model parameter estimation of a transient signal in noise, the methods which employ the cumulants defined and evaluated in different ways do produce results with different accuracies.

Our investigation shows that the proposed method based on a new definition of the fourth order cumulants does satisfy some optimality criteria. In particular, it is demonstrated that the symmetric fourth order cumulant sequence as defined here is independent of time, even though the underlying signal is non-stationary in nature.

In the accompanying paper, it is demonstrated that the method based on symmetric fourth-order moment/ cumulant can be applied for parameter estimation of signals in multiplicative and additive noise (Gaikwad et al. 2015). The concept of accumulated fourth-order moment has been extended to the concept of accumulated fourth-order cumulant for parameter estimation of non-stationary signals.
